# Massively Parallel Haplotyping on Microscopic Beads for the High-Throughput Phase Analysis of Single Molecules

**DOI:** 10.1371/journal.pone.0036064

**Published:** 2012-04-30

**Authors:** Jérôme Boulanger, Leila Muresan, Irene Tiemann-Boege

**Affiliations:** 1 Cell and Tissue Imaging Core, Centre National de la Recherche Scientifique, Institut Curie, Paris, France; 2 Radon Institute for Computational and Applied Mathematics of the Austrian Academy of Sciences, Linz, Austria; 3 Department of Knowledge-Based Mathematical Systems, Johannes Kepler University, Linz, Austria; 4 Institute of Biophysics, Johannes Kepler University, Linz, Austria; Natural History Museum of Denmark, University of Copenhagen, Denmark

## Abstract

In spite of the many advances in haplotyping methods, it is still very difficult to characterize rare haplotypes in tissues and different environmental samples or to accurately assess the haplotype diversity in large mixtures. This would require a haplotyping method capable of analyzing the phase of single molecules with an unprecedented throughput. Here we describe such a haplotyping method capable of analyzing in parallel hundreds of thousands single molecules in one experiment. In this method, multiple PCR reactions amplify different polymorphic regions of a single DNA molecule on a magnetic bead compartmentalized in an emulsion drop. The allelic states of the amplified polymorphisms are identified with fluorescently labeled probes that are then decoded from images taken of the arrayed beads by a microscope. This method can evaluate the phase of up to 3 polymorphisms separated by up to 5 kilobases in hundreds of thousands single molecules. We tested the sensitivity of the method by measuring the number of mutant haplotypes synthesized by four different commercially available enzymes: Phusion, Platinum Taq, Titanium Taq, and Phire. The digital nature of the method makes it highly sensitive to detecting haplotype ratios of less than 1∶10,000. We also accurately quantified chimera formation during the exponential phase of PCR by different DNA polymerases.

## Introduction

Knowledge about the combination of genetic markers on the same parental chromosome, known as the haplotype, has been extremely valuable to understand biological processes such as recombination, population migration and selection. As such, the human genome has been extensively genotyped by different genome initiatives to infer haplotypes from patterns of linkage disequilibrium [1], [2]. In the medical field, haplotyping has been used for mapping disease genes or identifying particular combinations of alleles that confer a greater susceptibility in complex traits. Haplotype information is also becoming quite relevant in functional biology since structural or polymorphic variations can have different phenotypic effects if found on the same or on the homologous chromosome (reviewed in [3]).

Haplotype information can be indirectly reconstructed in short regions by population-based phasing approaches that use information on linkage disequilibrium [4] or from next-generation sequencing data using mate-pair information or different insert-size libraries that link markers together [5]. Alternatively, unambiguous haplotype data can be generated by direct molecular haplotyping methods developed in the past decades. Typically, most of these approaches involve the physical separation of maternally and paternally derived genomic material either by dilution of single haploid cells such as sperm (known as sperm typing) [6] or by procedures separating individual chromosomes or large genomic pieces by fosmid cloning [7], microfluidic techniques [8] or dilution [9]. It is even possible to assess haplotypes directly on the DNA stretched on a slide and labeled with single fluorescent dyes by multicolor total internal reflection fluorescence microscopy [10].

All these previous methods provide haplotype information of large genomic regions, but the labor-intensiveness of the sample preparation limits the throughput. Efforts to improve the throughput include dilution of genomic DNA, followed by multiplex amplification of short polymorphic regions that are then genotyped achieving modest throughput [11]. Other high-throughput haplotyping methods rely on large starting amounts of DNA. Such methods include long range allele-specific PCR in which a certain combination of markers is preferentially amplified over the alternative alleles that form a mismatch with the 3′ end of the primers [12], [13]. This approach requires amplifying regions several kilobasas in size which can proof difficult. Alternatively, different genomic regions can be fused inside an emulsion compartment forming short haplotypes that can be easily characterized downstream [14], [15]; an ideal method to characterize the diplotype in different individuals and identify inversions or different isoforms [14].

Currently, haplotyping of single molecules has only a modest throughput or high-throughput haplotyping lacks single molecule resolution. There is no haplotyping method that combines both high-throughput and single molecule resolution. For this reason, it has been extremely difficult to characterize rare variants in large pools of wild types. In the medical field, such a haplotyping assay could identify whether mutations are in *cis* or *trans* which would improve considerably our understanding of tumorogenesis [16]. The early detection of cancers require a highly sensitive assay, that can detect mutant levels present roughly at 0.01% [17]. But so far, there is no suitable haplotyping method that can analyze tens of thousands single molecules in a feasible experiment. An initial development of a high-throughput single molecule haplotyping technology based on the amplification of DNA templates in an acrylamide matrix was attempted, but involved difficult amplification procedures in an acrylamide format and has not been developed further [18].

**Figure 1:**
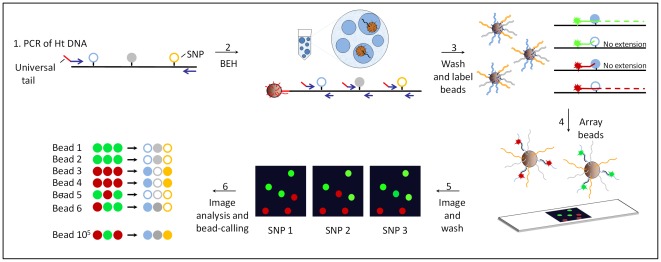
Schematics of bead-emulsion haplotyping (BEH). In step 1, a region of several kilobases containing several polymorphic sites is amplified from genomic DNA heterozygous for the tested SNPs. In step 2, single amplicons are hybridized to microscopic beads covered with a primer that has a universal tail common to the 5′ end of the multiplexing primers (shown in red). PCR products from 3 small regions containing the polymorphisms are produced within the aqueous compartment of an emulsion droplet and bound to the bead. In step 3, the beads are washed and labeled by allele-specific extensions of fluorescent probes specific for one of the polymorphic sites. In step 4, unextended probes are washed off and the fluorescent beads are arrayed on a slide. In step 5, the array is scanned with a microscope followed by subsequent washing, probing and imaging cycles to screen additional polymorphisms (up to 4 different alleles; 2SNPs can be analyzed simultaneously). In step 6, a series of imaging and data analysis steps are performed to assess the initial haplotype of ∼10^5^ molecules.

For the work presented here, we developed a high-throughput single molecule haplotyping method, called Bead-Emulsion Haplotyping (BEH) illustrated in Figure 1. Our approach is similar to the methods used in next-generation sequencing such as SOLiD™ Systems (Applied Biosystems) or 454 sequencing (Roche) in which single molecules are amplified on microscopic magnetic beads in an emulsion [19], [20], [21], [22]. So far, one serious limitation of bead-emulsion amplification has been the short size of the amplicons that can be produced on the beads [22]. The efficient amplification of up to 450 base pair products has been achieved using a Titanium Taq based protocol [19], but it is still very difficult to amplify longer regions with a good efficiency. This has hindered the use of this technology for haplotyping purposes given that usually polymorphisms are found at longer distances from one another. We have overcome this limitation by amplifying multiple independent regions regardless of the distance between polymorphisms. In our BEH approach, multiple polymorphic loci are amplified on microscopic beads in an emulsion, such that the recovered beads capture the phase of the initial single molecule. At the end of the reaction, the short PCR products attached to the beads are labeled with fluorescent probes and analyzed with an automated microscope as described here and previously [19]. The phase of the alleles in the original single molecule is then decoded from a series of images taken of the beads (see Figure 1). The throughput of BEH is similar to single loci amplification [19] and it is possible to study the phase of up to three polymorphisms separated by 5 kilobases in hundreds of thousand single molecules in parallel. We tested the robustness and performance of BEH by measuring the mutation rates of different PCR polymerases for which several different published values can be compared. We also analyzed the rate of chimera formation, a common PCR outcome creating false recombinants, that is usually ignored but contributes to a large fraction of PCR artifacts that are confounded as alternative splicing variants or new haplotype types (reviewed in [23]). Given the throughput and single molecule resolution of BEH, we accurately measured rates of chimera formation for polymerases with different proccessivities and proof-reading activities and we determined for the first time whether these properties have an effect on chimera formation in the early PCR cycles.

## Methods

### 1. Samples

Genomic DNA was obtained from anonymous human donors according to protocols approved by the Institutional Review Board of the University of Southern California. A signed patient consent form was obtained for all participants. DNA was extracted using the Gentra Puregene Cell Kit (Qiagen) with the addition of 24 µM DTT and 60 µg/mL proteinase K during the cell lysis step. Samples were genotyped by the Broad Institute Center for Genotyping and Analysis using the iPLEX (Sequenom) platform. Genotypes were confirmed with allele-specific PCR as described in [12].

### 2. Bead-emulsion haplotyping (BEH)

Previous to BEH, a region encompassing 2–4 SNPs (rs2837267, rs2837269, rs2299753, and rs2299754) on chromosome 21 was amplified from human genomic DNA. A 2733 bp amplicon was produced with Phusion Hot Start (Finnzymes) for most of the BEH experiments. The first 26 nucleotides of the different forward primers used in the amplification of genomic DNA correspond to a universal sequence present also at the 3′ end of the dual biotinylated primer attached to the bead's surface. Details of the PCR conditions employed to produce templates for BEH are described in Methods S1. A total of ∼3×10^6^ templates (amplicons) were hybridized to ∼10^7^ magnetic streptavidin coated Dynabeads M-270 (Invitrogen) as described by [12] such that based on the Poisson distribution only a small fraction of the beads are attached to more than one template. These multitemplate beads result in multicolored beads that could be easily eliminated during the analysis steps. The bead-emulsion amplification was carried out by mixing 150 µl of aqueous phase (1× Titanium Taq buffer, 8 mM MgCl2, 1 mM dNTPs, 9 µM of each forward primer, 0.05 µM of each reverse primer, and 2 U of Titanium Taq) with 800 ul oil-phase prepared with 116 mg Abil WE09 (Evonik Degussa), 580 µl TEGOSOFT (Evonik Degussa) and 160 µl light mineral oil (Sigma). Two to four different primer sets (forward and reverse) were added, each at the aforementioned concentration, such that each primer set amplified a small region of the template (70–100 base pairs). The aqueous and oil-components were emulsified in a cryovial with a PowerGen 125 homogenizer (Fisher Scientific) at speed level 2 for 50 seconds. The resulting emulsion was divided into small aliquots of 80–100 microliters and amplified in a standard thermocycler with an initial denaturation step of 2 minutes at 94°C followed by 55 cycles at 95°C for 30 seconds, 54°C for 15 seconds, and 68°C for 75 seconds. The beads were washed as described previously [19] and labeled using allele-specific extensions of Alexa coupled probes. The labeling was carried out either in a 50 microliter solution or directly on arrayed beads using HBW75 chambers (Sigma) by incubating the reaction for 2 minutes at 95°C, cooled for 5 minutes at 52°C, heated to 60°C for 5 minutes and 68°C for another 5 minutes, and then washed with 1E buffer [19] followed by two TE washes. Primer and probe details can be found in Methods S1.

### 3. Image acquisition and analysis

The labeled beads were arrayed and fixed with an acrylamide solution in a monolayer of 1 square centimeter on a Silane (Sigma) pre-treated microscope slide. The array was scanned with an automated inverted microscope (Zeiss Axio Observer.Z1) at a 20× magnification. Each fluorophore was captured with a 120 W mercury short arc lamp and individual filter cubes optimized to minimize crosstalk between the four different Alexa fluors [19]. At each raster position, 3 (respectively 5) images were captured of the arrayed beads with 12-bit 4K CCD camera: 1 bright field, and two (respectively four) fluorescent wavelengths. Approximately 280–300 raster positions were required to scan the array. The software Metamorph (MDS Analytical Technologies) was employed to control the microscope and convert the images into a list of intensity values for objects identified as beads. Specifically, the bright field image of each raster position was used to transform the grayscale image with a series of Metamorph morphology filtering and morphology analysis steps into a mask. Each mask consisted of a binary image with regions of interest (ROI) that defined the area of a bead. The mask was then employed to extract the intensity values for the defined ROI across the different fluorescent channels. Finally, a list of average pixel intensities for each ROI was compiled for each fluorescent channel used in the experiment. A more detailed explanation of these steps can be found in Methods S1 and Figure S2.

### 4. Imaging several SNPs

Up to 2 different polymorphisms labeled with 4 different Alexa dyes-A488, A532, A594, and A647 could simultaneously be screened. For some experiments we assayed more than two SNPs (4 fluorphores) or we screened SNPs labeled by the same fluorophore. In these experiments, we stripped the original fluorescent label by incubating the array at 94°C in TE and re-labeled the array using a HBW75hybridization chamber (Sigma) and an *in-situ* PCR block. The re-labeled array was scanned again such that images of different scans from the same array could be compared. Small misalignments between images of different scans caused by the manipulation of the slide were corrected by computing the normalized cross correlation matrix of pairs of images in Matlab (Mathworks). The x-y coordinates of the maximum of this matrix also corresponded to the x-y offset necessary to align the two images. For better time efficiency, the calculation of the cross correlation matrix was performed on a smaller central region that was size adjusted empirically to ensure sufficient overlap. These x-y offsets were then fed into Metamorph for the analysis of arrays scanned multiple times. A common mask was created for multiple scans that consisted of the intersection of ROI from the equivalent bright field images across scans. This common mask was then used to extract the intensity reads in the different fluorescent channels and scans.

### 5. Bead calling

The beads were classified into different clusters based on the signal intensity captured in different fluorescent channels. Beads were classified into one of four clusters: 00 (both channels are negative), 10 (first channel is positive), 01 (second channel is positive) and 11 (both channels are positive) based on the intensity values obtained for the two fluorescent channels used per SNP.

#### Normalization of the data

First, we normalized the data based on the mean and covariance of the cluster of the 00 class, given that most of the beads (∼90%) were empty (no amplification product). The parameter estimation of the 00 class can be influenced by the other classes and thus we implemented a robust approach to adapt for different amounts of beads in the other clusters. In short, the center of the cluster 00 was estimated using a mean-shift algorithm and a covariance matrix with a penalized likelihood approach [24]. For the penalized likelihood approach, we determine how much data should be taken into account in order to estimate the covariance matrix. The data was then ordered as a function of the Mahalanobis distance (distance between 2 random vectors considering the correlation between them) to the center of the cluster. An estimate and penalized likelihood criterion was computed for a series of growing data sets. The estimate corresponding to the minimum of the criterion was used to perform a normalization of the intensities.

#### Classification of the beads

Next, we modeled the intensities by a Gaussian Mixture Model (GMM), where each component of the GMM corresponds to a class. Additional constraints on the mixture parameters were added such as the symmetry and size of the clusters 10 and 01 in order to limit the search space and make the approach more robust. An expectation-maximization (EM) algorithm was applied [25] to estimate both the mixture parameters and the probability to belong to each class. Since the EM algorithm is known to be sensitive to initialization, a non-conservative classification was provided using a Bonferroni corrected threshold on MvA-plots used commonly in two-color microarray experiments [26]: M = log(R/G) vs A = log(RG), where R and G are the intensities of the two channels. If a component is almost empty at the initialization step, then the parameters of this component were not updated. If the probability to belong to a class was too low, beads could be discarded from a class using a predefined p-value. Results of the bead's classification for different SNPs were concatenated and reported as a user-defined sequence string. The bead-calling was performed in Matlab (Mathworks) through a graphical user-friendly interface. A more detailed description about the statistics can be found in Methods S1 and Figures S3, S4, and S5.

## Results

### 1. Multiplex amplification of up to three different polymorphisms

The amplification in emulsion on a solid phase, like microscopic beads, is restricted to short amplicons. This has hindered haplotyping SNPs distributed on genomic regions of at least several kilobases. Nevertheless, distant SNPs can be analyzed with bead-emulsion amplification using a multiplex approach as reported here. Multiplex PCR is a commonly employed technique but is tied to inherent problems such as competition between reactions rendering unequal amounts of PCR products or an overall reduction in PCR efficiency. We therefore assessed the performance of multiplexing in a bead-emulsion setting by comparing three parallel experiments differing in the number of amplified SNPs (1, 2 or 3 SNPs). For this purpose, we used a 2733 bp amplicon derived from genomic DNA heterozygous for SNPs separated by 800, 422 and 927 bp. We first analyzed whether the data output reproduces the input sequences. Our results show that the output sequence strings varied only minimally from the input ratio of one for each individual SNP. Moreover, the measured ratio of the expected haplotypes with 2 or 3 loci was very close to one throughout all our experiments (see Table 1 and Tables S1, S2, S3).

**Table 1:** Bead-emulsion haplotyping (BEH) targeting 1, 2, or 3 SNP regions.

	Number of beads^1^	Allelic ratio^2^	Informative beads^3^
**Monoplex BEH-SNP1**			
C-beads	34489	1.01	100.0%
T-beads	34120		
**Multiplex BEH-SNP 1+2**			
CA-beads	29131	1.02	80.8%
TG-beads	29802		
C0-beads	2295		6.7%
T0-beads	2625		
0A-beads	4784		12.4%
0G-beads	4259		
**Multiplex BEH-SNP 1+3+4**			
CTC-beads	30160	1.09	48.8%
TCT-beads	32935		
0CT-beads	9623		21.8%
0TC-beads	4804		
T0T-beads	3363		
C0C-beads	3944		
TC0-beads	3356		
CT0-beads	3134		
C00-beads	3198		
T00-beads	2531		
0C0-beads	18765		
0T0-beads	3349		29.4%
00C-beads	4016		
00T-beads	6224		

1 Number of beads counted for a specific sequence string in a total of 500,000 beads. The “0” in the sequence string represents an empty position for which only background florescence was recorded. Beads positives for more than one allele (two alleles per SNP) derived from multi-template reactions were removed from the data.

2 Ratio of alleles obtained for heterozygous DNA.

3 Sum of beads informative for the queried SNPs relative to the total number of beads with a product. The sum of different types of drop-outs (“0” in sequence string) are shown for the multiplex reactions.

**Figure 2:**
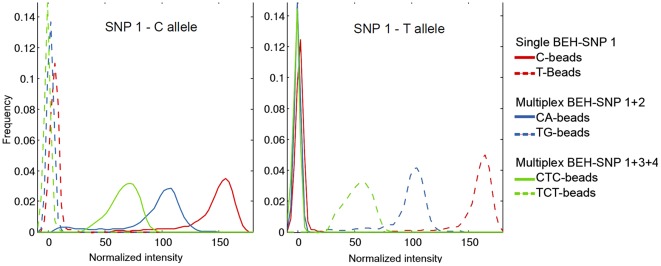
Distribution of the fluorescence intensity of the same target (SNP 1) common in all three experiments amplified with one, two or three targets. The intensity was normalized for the two fluorphores used per polymorphism. The high peak on the left represents empty beads without an amplification product.

We also examined whether multiplexing affects the unequal amplification of different targets . We observed that the amplification of more than one locus had an effect on target loss. In a reaction with two SNPs, approximately 20% of the beads amplified only one or the other SNP, but not both (see Table 1). The percentage of target loss increased to ∼50% when simultaneously amplifying three SNPs. Of those 50%, half of the beads had amplified only two out of three SNPs and the other half had amplified only one of the three SNPs. Multiplexing 4 SNPs resulted in very few beads informative for all four loci and thus was not explored further (data not shown). Comparable results were obtained in different experiments that amplified different SNP combinations or target lengths (see Table S1). We also observed a decrease in the intensity of the fluorescent signal with increasing numbers of multiplex reactions. Given that the average fluorescence intensity of the beads is proportional to the amount of amplified product and thus an indicator of PCR efficiency, we compared the fluorescence intensity of the beads amplified with 1, 2 or 3 SNPs. For the same fluorophore and locus, we observed a decrease in intensity with an increasing number of multiplex reactions. In other words, when amplifying two SNPs the average fluorescent signal of the beads was reduced compared to beads amplified only with one SNP. Similarly, the amplification with three multiplex reactions (3 SNPs) rendered dimmer beads than with 2 multiplex reactions (2 SNPs). Since not all the beads with a PCR product had the same intensity, the reduction in fluorescence was observed as a shift in the intensity distribution (see Figure 2). The reduction in intensity had the effect that the difference between beads with a product and empty beads became smaller. Interestingly, beads with a target loss of one locus had a similar intensity distribution than monoplex beads (see Figure S1).

### 2. Haplotyping of polymorphisms on 5 kb templates

**Figure 3:**
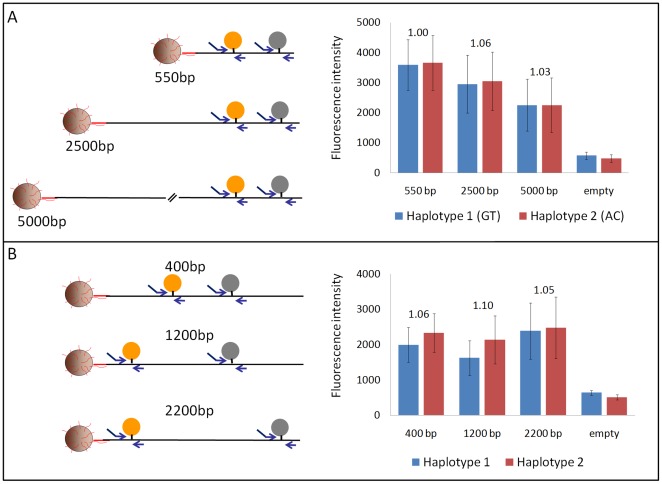
BEH on different template lengths and SNP positions. The left panel is a schematic of the location of the amplified SNP region relative to the template. The right panel represents the sum of the fluorescence intensity obtained for both alleles for the possible haplotypes. The error bars are the standard deviation of the fluorescence intensity. Shown above each pair of haplotypes is the observed allelic ratio of the captured haplotypes. A. Assay of the same two SNPs (422 bp apart) on templates of different lengths. B. Assay of different SNPs pairs 400, 1200, and 2200 base pairs apart on the same 2733 bp template.

To explore the performance of the method using different starting template lengths and SNP positions, we assayed the same SNPs forming a GT or AC haplotype in a series of templates ranging from 550 to 5000 base pairs. We then compared the sum of the average fluorescence intensity obtained for the two SNPs measured in the GT beads or AC beads. We show in Figure 3A that the average fluorescence intensity is similar among beads regardless of the length of the starting template. Moreover, for each of the different template lengths the ratio of sequence strings was again very close to the expected input ratio of one. To test whether the position of a SNP had an influence on its amplification efficiency, we assayed different SNPs on the same 2733 bp template. We show in Figure 3B that again the sum of the average fluorescence intensity is comparable between haplotypes formed by different SNP pairs and the observed allelic ratios were very close to the expected ratio of one. Moreover, there is not a preferential amplification of 5′ regions over 3′ regions, the former being closer to the primers on the bead's surface to start the amplification on the bead. In addition, the observed target loss was similar among SNPs at different distances and different template lengths (see Table S1). Our results clearly show that the multiplex amplification efficiency of different SNPs regions is independent from the SNP's position on the template and also from the template length. This was not unexpected given that each reaction is seeded with a very low primer concentration in solution to jumpstart the emulsion PCR.

### 3. Sensitivity of BEH

Given the large number of single haplotypes that can be examined with BEH, we assessed the sensitivity of our method by characterizing PCR polymerase error-rates that can be compared to the literature as done previously [20]. We measured the mutation rate of different PCR polymerases with BEH by assessing the haplotypes formed by two SNPs (one homozygous and the other heterozygous) in a 422 bp region. This set-up allowed us to screen new mutations at the homozygote SNP and control at the same time for the correct haplotype ratios using the heterozygote SNP. Genomic DNA was amplified for 35 cycles with four commercially available thermostable polymerases (Phusion, Platinum Taq, Phire, and Titanium Taq). The fraction of mutants was measured by counting the beads with a thymidine instead of the expected cytidine in the amplified products. The lowest mutation fraction was observed for Phusion (Finnzymes) with 1.0×10^−5^ mutants per wild type equivalent to an error rate per cycle of 3.0×10^−7^ per nucleotide. The highest mutation fraction was observed for Platinum (Invitrogen), a Taq equivalent with 9.5×10^−4^ mutants per wild type (or an error rate per cycle of 2.7×10^−5^). Titanium Taq (Clonetech) and Phire (New England Biolabs), both enzymes with a higher proccessivity, presented a mutation fraction of 1.7×10^−4^ (error rate per cycle of 5.1×10^−6^) and 2.2×10^−4^ (error rate per cycle of 6.2×10^−6^), respectively. Finally, for all the haplotypes the observed ratio was very close to one (see Table S2). These results reflect that BEH is sensitive enough to detect haplotypes that are as rare as 1∶10,000 or less.

### 4. Rates of chimera formation during PCR

**Figure 4:**
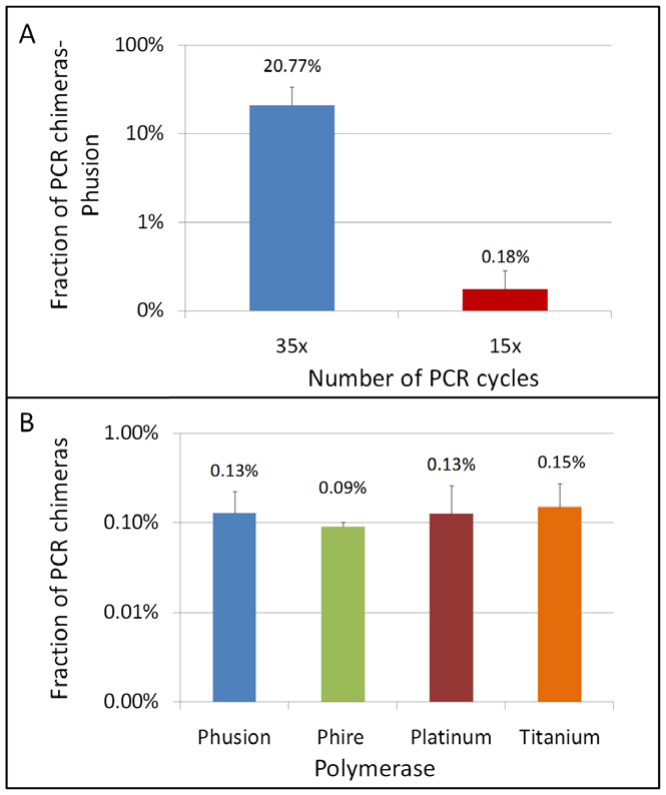
Assessment of chimeras formed during PCR. A. Comparison of the fraction of false recombinants measured in 400–2200 bp templates amplified with Phusion polymerase using either 35 or 15 PCR cycles. The fractions are averages estimated from at least 5 independent experiments using different template lengths (error bars are the estimated standard deviation). B. Fraction of false recombinants measured in 422 base pair PCR products amplified for 15 cycles by different polymerases. The fractions are averages estimated from three independent experiments and error bars are the estimated standard deviations.

We measured rates of chimera formation at high accuracy for polymerases with different proccessivities and proof-reading activities to test whether these properties have an effect on chimera formation. In order to measure the frequency of chimera formation, we amplified different sized products in the range of 400–2200 base pairs using genomic heterozygote DNA for at least two SNPs. Then with BEH, we evaluated the number of chimeras present in the amplicons produced. All of the observed recombinants or chimeras are PCR artifacts given that we used blood DNA as our source of genomic DNA for which no recombination products are known. Using Phusion, the PCR polymerase with the highest reported fidelity, we observed that one out 500 amplicons (0.2%) was a chimera or false recombinant (chimera rate per cycle of 1.2×10^−4^) when producing templates with 15 PCR cycles. In comparison, one out of every 5 amplicons was a chimera or false recombinant (21% of chimeras) when producing templates with 35 PCR cycles (see Figure 4A). This is an chimera rate per cycle of 5.9×10^−3^. Similar chimera fractions were also observed with Phire for different product lengths (422 bp to 5000 bp) indicating that chimera formation is a common phenomenon independent of product length (see Table S3). In our system, the plateau phase was reached at 35 cycles with already 2 ng (equivalent to 600 copies) of starting human genomic DNA as we monitored with real-time PCR. Comparing the values for chimera rate formation per cycle (1.2×10^−4^ and 5.9×10^−3^) using 15 or 35 cycles, respectively, it is clear that chimera production occurs at a much lower rate during the exponential phase of the PCR than at the plateau phase.

To examine whether the proof-reading activity or the proccessivity of the PCR polymerase has an effect on chimera formation during the exponential phase of PCR, we compared amplicons produced by Phusion (Finnzymes), Phire (Finnzymes), Platinum (Invitrogen), and Titanium (Clonetech). Phusion is a polymerase with proof-reading activity and with the highest known fidelity, and Titanium Taq and Phire have improved proccessivities compared to Platinum. We amplified for 15 cycles genomic DNA heterozygous for two different SNPs producing a 422 base pair fragment using the recommended conditions by the manufacturer. Our results show that the frequency of chimeras is independent of the proof-reading activity or proccessivity of the used PCR polymerases. For all four different polymerases (Phusion, Phire, Platinum, and Titanium) we obtained similar frequencies of chimeras ranging from 0.09% to 1.5% recombinants per measured haplotype (see Figure 4B) suggesting that chimera formation is independent of the polymerase's properties during the exponential PCR phase.

## Discussion

### Capability of BEH

We have developed a methodology capable of analyzing haplotypes spanning a 5 kb region in hundreds of thousands single molecules in one experiment. This far exceeds the capability of next-generation sequencing approaches that have a similar throughput but achieve a maximum continuous stretch of ∼400 bp with 454 Sequencing. A similar method that amplifies single molecules by multiplex PCR followed by allele-specific primer extension has been used previously achieving ∼1,000 haplotypes a day [11]. The microscopic format of each PCR reaction in BEH allows screening ∼100,000–500,000 molecules in one experiment in half a day. As with next-generation sequencing protocols, the miniaturization of the reactions decreases drastically the costs and each BEH experiment costs no more than 10 regular real-time PCR reactions involving fluorescent probes. Moreover, scoring of the beads can be done with any automated fluorescent microscope or alternatively with a multi-colored flow cytometer (if only 2 SNPs are assessed). Our haplotyping approach based on the amplification of multiple polymorphic loci on microscopic beads in an emulsion is robust to haplotype up to 6 different alleles (3 SNPs) distributed over 5 kilobases in single molecules. The analysis of more SNPs is limited by the number of targets that can get labeled and the degree of target loss. It is unlikely that the observed target loss is a result of an overall reduction in fluorescence intensity, since the target loss is random and there is no over-representation of a particular target. Target loss is a common problem when amplifying low copy numbers and similar fractions of target loss (25–50%) have been observed even in a standard 4–6 Plex PCR starting with a few copies [27]. Target loss not only reduces the amount of usable data but could create a series of artifacts if coupled with allelic drop-out. Allelic drop-out is known to cause false haplotypes if only one of the two alleles gets amplified in a sample with more than one starting template [28]. The target loss observed in our experiments might be also related to the heterogeneous compartment size characteristic of emulsions formed by stirring or homogenization. Droplet formation with microfluidics can produce uniform and 100 fold larger emulsion compartments that result in a comparable PCR efficiency between droplets [29]. Whether larger and homogeneous droplets can increase the number of multiplexing reactions in a bead-emulsion setting would need further testing.

In our assay, we tested templates as long as 5 kb, but theoretically even longer templates could be used. Template size is limited by the volume of the emulsion compartment. It was estimated that the typical size of an emulsion compartment is 10^−4^ nl (5 µm in diameter) [21], [30] or 2.7×10^−2^ nl (30 µm in diameter) in our experiments. These compartments easily accommodate 5 kb and longer templates given the flexible nature of the DNA molecule; although, the length is also restricted by the state of the fragmentation of the initial genomic DNA. Moreover, a denatured DNA strand could form random coils compacting the DNA even more. The compartment sizes of our emulsion procedure would be large enough to even hold human sperm heads (5 µm by 3 µm) and in theory it would be possible to haplotype SNPs throughout the human genome if sperm can be amplified without any pre-treatment, which usually requires an alkaline lysis step in single sperm typing assays [31]. It is also possible to amplify genomic DNA directly from cells within an emulsion and the successful amplification of single bacterial cells in emulsion has been reported beforehand using large compartment sizes (4 nl) that can dilute any inhibitor released by the cell lysis [29].

Finally, although BEH primers and probes are specific for each assay, the system is flexible enough to analyze different regions or polymorphisms without lengthy optimizations. The amplification of small independent PCR products on the beads is fairly robust and independent of the SNP's position or template length as we have shown. Moreover, given that a universal primer-system is used, primers with similar melting temperatures can be designed anywhere within 150 bases around the polymorphisms unless a SNPs are located within an unusual genomic region (e.g. low complexity DNA). Even the labeling step is quite robust given that it relies on allele specific extensions of labeled probes which is very specific in contrast to hybridizations.

### Sensitivity of the assay

BEH is also a highly accurate and sensitive method given its high-throughput and single molecule nature. The sensitivity of the method was validated by measuring the mutation rates of different PCR polymerases for which several different published values were compared. The error rates per cycle estimated from our experiments for Phusion and Platinum Taq are comparable to very similar values of 4.2×10^−7^ and 4.2 to 1.9×10^−5^, respectively, reported in the literature [20] and commercial providers; although, we believe that our measured error fractions are underestimates since only one of the possible three “mutant” nucleotide was assessed. Although, no precise description of the error rate per cycle was available for Titanium and Phire, commercial providers claim a 2× higher fidelity compared to Taq. We measured a 1.5–3× lower mutation rate confirming again that BEH is a highly sensitive and accurate method.

It is difficult to assess the limit of the sensitivity for BEH. The 1∶10,000 mutation rate we measured were nucleotide misincorporations introduced during 35 cycles of PCR. Errors that occurred during the emulsion PCR step of BEH rendered multi-template beads that were identified as multicolored beads and removed from the analysis. Lowering the number of cycles of the initial PCR by half improves the sensitivity several fold (unpublished data). Predicting the number of mutations that accumulate during the initial PCR is difficult given that this is a complex process depending on the exponential or plateau phase of the amplification, the different amplification efficiencies of heterogeneous molecules in the pool, and the initial quality of the DNA. Thus, the initial number of PCR cycles required to achieve a given sensitivity will depend on each particular system. Alternatively, genomic DNA could be used as starting material as discussed previously.

### Chimera formation

Another important error source during PCR is template switching that render false recombinant DNA molecules known as chimeras. The formation of chimeras is a serious problem that can confound research results especially if a population of highly similar sequences are analyzed such as microbial samples [32], [33], [34] or the MHC [35], [36]. Moreover, chimeras are quite common and our results indicate that as much as a quarter of the PCR products are chimeras of two flanking polymorphisms if the amplification reached the plateau phase. Similar values were observed for the plateau phase in the literature ranging from 24% in a 198 bp fragment amplified from genomic DNA [35], 36–45%-in a 670 bp fragment amplified from 8 different clone haplotypes [37], 10–13% in a 4.5 kb amplified from a plasmid [38] to 5% in a 275 bp fragment amplified from a plasmid [39]. Chimera formation can be a consequence of truncation products acting as primers to the homologous templates [39], [40] or of template jumping in which the polymerase switches synthesis to another pre-existing homologous template [23], [40].

In the last years, a large effort has been invested to understand the different factors affecting chimera formation in order to reduce this PCR artifact. Different studies have provided conflicting data about the effect of the quality and length of starting DNA, elongation time, Taq proccessivity and proofreading activity in the frequency of chimera formation. In this work we took advantage of our highly sensitive BEH method to investigate chimera formation at PCR conditions well before the plateau phase, given that these conditions are more representative, but are difficult to quantify with the known available methods. The chimera formation seems to be much higher near or at the plateau phase [33], [34], [41], [42], [43], but so far samples sizes have been too small to provide a robust measure of chimera formation in early cycles. We observed a ∼50 fold difference in the rate of chimera formation between the exponential and plateau phase. Regarding proof-reading activity or proccessivity of PCR polymerases, our results show that proof-reading enzymes such as Phusion or polymerases with enhanced proccessivity (Phire and Titanium) do not reduce the frequency of chimera formation compared to standard polymerases (Platinum) during the exponential phase of the PCR. This conflicts with studies reporting a lower chimera formation for Phusion [37]. Similarly, proccessivity enhancing PCR factors such as betaine and DMSO also have been reported to decrease chimera formation [44]. It is possible that the role of proccessivity and proof-reading is more complex than assumed and could have different effects in chimera formation during different stages of PCR. Eliminating chimeras could be achieved by avoiding large mixtures of different DNA molecules that become scrambled during amplification. Amplifying genomic material in a single molecule format using a water/oil emulsion as described by Williams et al [45] could be the best resource to effectively reduce chimeras especially in applications with limited DNA material requiring many PCR cycles.

### Applications of BEH

So far there is no haplotyping method that combines both high-throughput and single molecule resolution of BEH. Although, BEH can only target pre-determined regions, the method is quite powerful to screen candidate regions associated with a certain disorder or phenotype in much more detail and could be employed in very different biological scenarios as exemplified next. In the context of cancer research, BEH would be ideal to target a known mutation and its distribution on the two homologue given that mutations in different homologues can affect gene function as reviewed by [3]. Associating a mutation to a heterozygous marker can also be used to infer the biological process propagating the mutation as has been done previously [46]. In the context of association studies, phasing a disease-causing variant with a genetic marker considerably increase the accuracy of the mapping as has been shown in family based genome sequencing studies [47]. Yet, for population or case-control studies the costs for directly inferring the phase are still prohibitive and phasing relies on statistical methods that are not always reliable. BEH could haplotype large populations or case-control cohorts of candidate markers and associated disease variants. The digital nature of the method provides an enormous accuracy even for very small differences in haplotype ratios in large sample sizes. In similar lines, BEH could easily verify distortions in allelic transmission inferred from genotype data and discover underlying biological processes that might be driving the distortion. In other applications, BEH is not limited to polymorphisms. The method can also be adapted for DNA methylation and provide information on methylation patterns and their associated alleles in cases where large sample sizes need to be screened. Knowledge of parent of origin of certain imprinted regions has been relevant for example in type 2 diabetes [48].

### Conclusions

We showed that BEH is a high-throughput haplotyping method with single molecule resolution. To our knowledge, this is the first time haplotypes of 3 SNPs over a 5 kb region of hundreds of thousand single molecules can be obtained in a single experiment in a short period of time. With BEH we can accurately characterize haplotypes in complex mixtures,quantify minor haplotype variants, and associate phase information with mutations which could play an important role especially if phase information is pertinent to elucidate diverse biological processes. As such, this method could have important applications in the accurate assessment of haplotype variants and capture the haplotype diversity in samples of different flora, cancer tissues or larger cohorts screened in clinical settings.

## Supporting Information

Methods S1 Details on PCR conditions used in different experiments such as primer sequences, amplification conditions, etc. This section also includes a extensive description on the data analysis and statistics used.
(DOCX)

Figure S1 **Intensity distributions of allelic dropouts compared to multiplex amplification.** Distribution of the fluorescence intensity of the beads amplified with one or two targets. The intensity was normalized for the two fluorphores used per polymorphism.
(EPS)

Figure S2 **Schematics of the bead analysis to extract the beads' intensities from the pixel information of an image.**
(EPS)

Figure S3 **Distribution of average intensities of two fluorescent channels.** The red, green, blue and pink cluster represent the 00, 10, 01, and 11 class respectively. It can be observed that it is difficult to delimit the 00 from the other 3 classes.
(TIFF)

Figure S4 **MvA plot with normalized intensities of two fluorescent channels.**
(TIFF)

Figure S5 **Normalized intensities of two fluorescent channels with the number of beads estimated for each of the four clusters.**
(TIFF)

Table S1 Target loss measured in the amplification of two targets using different template lengths and SNP combinations. ^1^Number of beads counted for a specific sequence string. The “0” in the sequence string represents an empty position for which only background florescence was recorded. Beads positives for more than one allele (two alleles per SNP) derived from multi-template reactions were removed from the data. ^2^Ratio of alleles obtained for heterozygous DNA ^3^Sum of beads informative for the queried SNPs relative to the total number of beads with a product.
(DOCX)

Table S2 Mutation rate of 4 different commercially available thermostable polymerases. The data was obtained amplifying a 422 bp template over 35 cycles that was then assayed by BEH targeting two loci. The fraction of mutants was estimated based on the number of beads with a thymidine instead of the expected cytidine. The error rate per cycle was estimated based on 35 cycles.
(DOCX)

Table S3 Chimera formation in different product lengths. Amplicons were produced using Phusion in 35 cycles of PCR.
(DOCX)
