# Supplementary material for: Massively Parallel Haplotyping on Microscopic Beads for the High-Throughput Phase Analysis of Single Molecules
Source: PLoS One. 2012 Apr 30;7(4):e36064. doi: 10.1371/journal.pone.0036064 (PMC3340404; doi:10.1371/journal.pone.0036064)
Supplement: Methods S1 — Details on PCR conditions used in different experiments such as primer sequences, amplification conditions, etc. This section also includes a extensive description on the data analysis and statistics used. (DOCX) [file pone.0036064.s001.docx]

**Massively parallel haplotyping on microscopic beads for the high-throughput phase analysis of single molecules**

Jérôme Boulanger^1,2^, Leila Muresan^3^, and Irene Tiemann-Boege^4^

^1^Cell and Tissue Imaging Core, Centre National de la Recherche Scientifique, Institut Curie, Paris, France

^2^Radon Institute for Computational and Applied Mathematics of the Austrian Academy of Sciences

^3^Department of Knowledge-Based Mathematical Systems, Johannes Kepler University, Linz, Austria

^4^Institute of Biophysics, Johannes Kepler University, Linz, Austria

**SUPPLEMENTARY MATERIALS CONTENTS.**

**SUPPLEMENTARY Methods**

1. PCR, primers and probe information.

2. Image analysis.

3. Bead calling

**SUPPLEMENTARY Figures.**

Supplementary Figure 1. Intensity distributions of allelic dropouts compared to multiplex amplification

**SUPPLEMENTARY Tables**

Supplementary Table 1. Target loss measured in the amplification of two targets using different template lengths and SNP combinations

Supplementary Table 2. Mutation rate of 4 different commercially available thermostable polymerases

Supplementary Table 3. Chimera formation in different product lengths.

**1. PCR, Primers and probe information.**

***PCR conditions to produce templates for Bead Emulsion Haplotyping***

- **2733 bp product:**

F-2733 bead 5‘-*ACA CCA AAC TAC AAA ATC ACT CAT TA* AAC AGT GTG GGC TGG ACA TT -3‘

R-754 5' TTGGTTTGATATTTAATTTGGTGAGA 3'

**Phusion** amplification conditions (50ul reactions): 1x buffer HF, 200µM dNTPs, 0.5µM Forward primer, 0.5µM Reverse primer, 0.5U Phusion HotStart (Finnzymes), and 50ng genomic DNA.

**Phire** amplification conditions (50ul reactions): 1x Phire reaction buffer, 200µM dNTPs, 0.5 µM forward primer and 0.5µM reverse primer, 0.5U Phire Hot Start DNA Polymerase (Fynnzymes), and 200ng of genomic DNA of a heterozygous donor, except if stated otherwise.

| **Temperature** | **Time** | **Cycle No.** |
| --- | --- | --- |
| 94˚C | 2 min | 1x |
| 94˚C | 15 sec | 15x or 35x |
| 60˚C | 30 sec |  |
| 72˚C | 80 sec |  |
| 72˚C | 5 min | 1x |

***PCR conditions used to produce different amplicon lengths:***

Amplification conditions (50ul reactions): 1x buffer HF, 200µM dNTPs, 0.5µM Forward primer, 0.5µM Reverse primer, 0.5U Phusion HotStart (Finnzymes), and 50ng genomic DNA.

- **550 bp product:**

F-600 (bead) 5‘-*ACA CCA AAC TAC AAA ATC ACT CAT TA* GAG GCA GTC CTG CAT TTT GT -3‘

R-753 5' CTACTGGCCACATCTAAGGAG 3'

| **Temperature** | **Time** | **Cycle No.** |
| --- | --- | --- |
| 98˚C | 30 sec | 1x |
| 98˚C | 5 sec | 35x |
| 58˚C | 5 sec |  |
| 72˚C | 15 sec |  |
| 72˚C | 2 min | 1x |

- **2500 bp product:**

F-2500 (bead) 5‘-*ACA CCA AAC TAC AAA ATC ACT CAT TA* ggt tgg ttc ctt ctg agc ac -3‘

R-753 5' CTACTGGCCACATCTAAGGAG 3'.

| **Temperature** | **Time** | **Cycle No.** |
| --- | --- | --- |
| 95˚C | 2 min | 1x |
| 95˚C | 15 sec | 35x |
| 58˚C | 15 sec |  |
| 72˚C | 80 sec |  |
| 72˚C | 2 min | 1x |

- **5000 bp product:**

F-5000 (bead) 5‘-*ACA CCA AAC TAC AAA ATC ACT CAT TA* TCC GTT GGA ATC AGA GAT CC -3‘

R-753 5' CTACTGGCCACATCTAAGGAG 3'

| **Temperature** | **Time** | **Cycle No.** |
| --- | --- | --- |
| 95˚C | 2 min | 1x |
| 95˚C | 15 sec | 35x |
| 58˚C | 150 sec |  |
| 72˚C | 160 sec |  |
| 72˚C | 2 min | 1x |

***Conditions for testing chimera formation by different PCR polymerases***

**518 bp product:**

F-269 (bead) 5‘-*ACA CCA AAC TAC AAA ATC ACT CAT TA* AGA TAT GCG GAT TCG TGT TTT TC -3‘

R-753 5' CTACTGGCCACATCTAAGGAG 3'

- **Phusion** (50ul reactions): 1x Buffer HF, 200µM dNTPs, 0.5 µM forward primer and 0.5µM reverse primer, 0.5U Phusion Hot Start DNA Polymerase (Fynnzymes), and 50ng of genomic DNA of a heterozygous donor.

| **Temperature** | **Time** | **Cycle No.** |
| --- | --- | --- |
| 98˚C | 30 sec | 1x |
| 98˚C | 5 sec | 15x |
| 58˚C | 5 sec |  |
| 72˚C | 15 sec |  |
| 72˚C | 2 min | 1x |

- **Phire** (50ul reactions): 1x Buffer HF Phire, 200µM dNTPs, 0.5 µM forward primer and 0.5µM reverse primer, 0.5U Phire II Hot Start DNA Polymerase (Fynnzymes), and 50ng of genomic DNA of a heterozygous donor.

| **Temperature** | **Time** | **Cycle No.** |
| --- | --- | --- |
| 98˚C | 30 sec | 1x |
| 98˚C | 5 sec | 15x |
| 58˚C | 5 sec |  |
| 72˚C | 15 sec |  |
| 72˚C | 2 min | 1x |

- **Platinum** (50ul reactions): 1x Buffer Platinum, 200µM dNTPs, 0.5 µM forward primer and 0.5µM reverse primer, 0.5U Platinum Taq (Invitrogen), and 50ng of genomic DNA of a heterozygous donor.

| **Temperature** | **Time** | **Cycle No.** |
| --- | --- | --- |
| 94˚C | 2 min | 1x |
| 94˚C | 15 sec | 15x |
| 58˚C | 15 sec |  |
| 72˚C | 30 sec |  |
| 72˚C | 2 min | 1x |

- **Titanium** (50ul reactions): 1x Buffer Titanium, 200µM dNTPs, 0.5 µM forward primer and 0.5µM reverse primer, 0.5U Titanium Taq (Clonetech), and 50ng of genomic DNA of a heterozygous donor.

| **Temperature** | **Time** | **Cycle No.** |
| --- | --- | --- |
| 94˚C | 2 min | 1x |
| 94˚C | 15 sec | 15x |
| 58˚C | 15 sec |  |
| 72˚C | 30 sec |  |
| 72˚C | 2 min | 1x |

**Primers and probes for BEH:**

- **Bead-primer:**

R-Bead Primer 5' /52-Bio//iSp9/TAT GTC TTT CTC TCA CAT AA *ACA CCA AAC TAC AAA ATC ACT CAT TA 3'*

52-Bio = 5' Dual biotin

iSp9 = Internal Spacer 9

The nucleotides in italics are a universal sequence present also at the 5' end of the forward primers used to amplify genomic DNA.

- **Multiplex primers:**

The primer name denotes the last 3 digits of the SNP's rs number that is amplified by the primer set.

R-267 AAGACCCAAAGGCTGAAATTCT

F-267 (bead) *ACA CCA AAC TAC AAA ATC ACT CAT TA* ATT CTC TTA CCA TAA AGG TCA GGA

R-269 GCAGCATCTCGACAAGCAGTT

F-269 (bead) *ACA CCA AAC TAC AAA ATC ACT CAT TA* AGA TAT GCG GAT TCG TGT TTT TC

R-753 CTACTGGCCACATCTAAGGAG

F-753 (bead) *ACA CCA AAC TAC AAA ATC ACT CAT TA* ACG CTG TCA CTG ATG GAT AA

R-754 TTGGTTTGATATTTAATTTGGTGAGA

F-754 (bead) *ACA CCA AAC TAC AAA ATC ACT CAT TA* AGC CTC CTT ATT CCC TAA CCC TTA

**Bead-emulsion haplotyping conditions:**

| **Temperature** | **Time** | **Cycle No.** |
| --- | --- | --- |
| 95˚C | 2 min | 1x |
| 95˚C | 30 sec | 55x |
| 54˚C | 15 sec |  |
| 68˚C | 75 sec |  |
| 68˚C | 5 min | 1x |

- **Allele-specific extension probes:**

Asterisk denote phosphorothioated DNA bases:

SNP-267-C 5' /Alexa 488/ATT CTG CCT CAA ATA CT*A* C*G 3'

SNP-267-T 5' /Alexa594/ATT CTG CCT CAA ATA CT*A* C*A 3'

SNP-269-A 5' /Alexa594/CAGTTCCCTAGATGGAT*C*A*T 3'

SNP-269 -G 5' /Alexa488/CAGTTCCCTAGATGGAT*C*A*C 3'

SNP-753-T 5' /Alexa532/TGGACCCAGCAG*C*C*A 3'

SNP-753-C 5' /Alexa647/TGGACCCAGCAG*C*C*G 3'

SNP-754-C 5' /Alex532/ttGTTGGACTGG*G*C*G 3'

SNP-754-T 5' /Alex647/ttGTTGGACTGG*G*C*A 3'

**2. Image analysis.**

The automated fluorescent microscope takes 3-5 images (1 bright field, and two or four) fluorescent wavelengths) at each raster position. The resulting files are 12 bit monochrome images. The pixel's value in a monochrome image is represented by a certain gray-scale palette which ranges for a 12-bit image from 0-4096. The maximum number of grays in a 12-bit image is 2^12^ = 4096. The pixel gray-scale is correlated with the amount of emitted light by the fluorophore. Thus, grayscale values can be used as a measure of light intensity which in turn correlates with the number of fluorophores or PCR product on the beads. Approximately 20-30 pixels define the area of a bead and the average gray scale value of these pixels of the total bead area is reported as the intensity of the bead.

In order to establish the area of a bead or region of interest (ROI), we use a series of Metamorph morphology filtering and morphology analysis steps to convert the bright field image into a binary image (only white or black pixels). The white pixels in the binary image represent the beads and can be easily transformed into a mask with ROI defining each bead. The mask is used to extract the gray scale values of each ROI across different fluorescent images for the same raster position. This results in a list of objects with an average intensity measured across the different fluorescent channels. In Figure S2 we represent the workflow of these different steps.

**2. Bead calling.**

Two different fluorophores are used to identify the allele at a given locus. The average intensity for each bead in the different fluorescent channels is then compared to classify the beads into four different clusters. There are four possibilities: background fluorescence in both channels associated with a low average intensity for both channels (00), fluorescence in one channel but not the other associated with a high intensity in one channel and a low intensity in the other (01 or 10), or fluorescence in both channels associated with a high average intensity in both channels. Ideally, the clusters should be well separated, especially if the difference in average intensity is large. In practice, the intensity difference of the majority of the beads is 5-10 fold but a fraction of the beads lie in between clusters making it difficult to demarcate a cluster. In Figure S3 we illustrate the distribution of average intensities of the two fluorescent channels. It can be observed that it is difficult to delimit the 00 from the other 3 classes.

A simple MvA-plot commonly used in two-color microarray experiments where R and G are the intensities of the two channels (M=log[R/G] versus A=log[RG]), does not perform very well (see Figure S4) with many beads being mis-assigned as 01 instead of 00, skewing the ratio of 10 and 01 class.

In order to improve the classification of the beads, we model their intensity distribution by a mixture of Gaussian such that each cluster is associated to a Gaussian distribution. This model is able to measure the possible dependency between channels and therefore can be used for the classification process. In order to estimate the parameters of the model defined by the mean and the covariance matrix of each class as well as the proportions of each class, we use the classical Expectation-Maximization (EM) algorithm. At each step, the algorithm computes the probability of a data point to belong to a class given some initial parameters for the classes and then the algorithm updates the parameters of the class using maximum likelihood estimators. However, given that this approach is sensitive to initialization, we use the following initialization step based on the robust estimation of cluster 00:

1. We pre-process the data by robust estimation of the parameters of the cluster 00 and then normalize the entire data, based on the previously estimated location and scale parameters. The location of the cluster is estimated using a mean-shift algorithm that is able to find the mode m of the dataset. The mean-shift is an iterative method and each iteration k is expressed as:

m^k+1^ = sum_i_ ( exp(-(x_i_-m^k^)²/2s²) x_i_ ) / sum_i_ ( exp(-(x_i_-m^k^)²/2s²) )

where m is the mode, s is the scale of the kernel, x_i_ are the data points indexed by i, and k are the iterations.

2. Given this location estimation, the covariance matrix is performed by using a penalized maximum likelihood approach. The data are sorted as a function of their distance to the estimated mode. A subset of k closest data points are used to estimate a first covariance matrix, then the data are scaled using this new covariance matrix C_k_ and are then re-ordered. At each iteration the criterion J is computed by:

J=log(det(C)) - c1 log(k/n)+c2 k/n

The value of C_k_ minimizing J_k_ is then used as final scale estimate

3. After normalizing the data with the estimated parameters, we initialize the EM algorithm with the classification result obtained from an MvA-plot analysis with a Bonfferoni corrected threshold.

A plot of the bead distribution after the implementation of our algorithm is shown in Figure S5. As can be noted, after the analysis the ratio of the 10 and 01 class is very close to the expected ratio of one given that the locus under investigation was heterozygous, confirming the proper implementation of the analysis.
