# Supplementary figures and images for: Massively Parallel Haplotyping on Microscopic Beads for the High-Throughput Phase Analysis of Single Molecules
Source: PLoS One. 2012 Apr 30;7(4):e36064. doi: 10.1371/journal.pone.0036064 (PMC3340404; doi:10.1371/journal.pone.0036064)

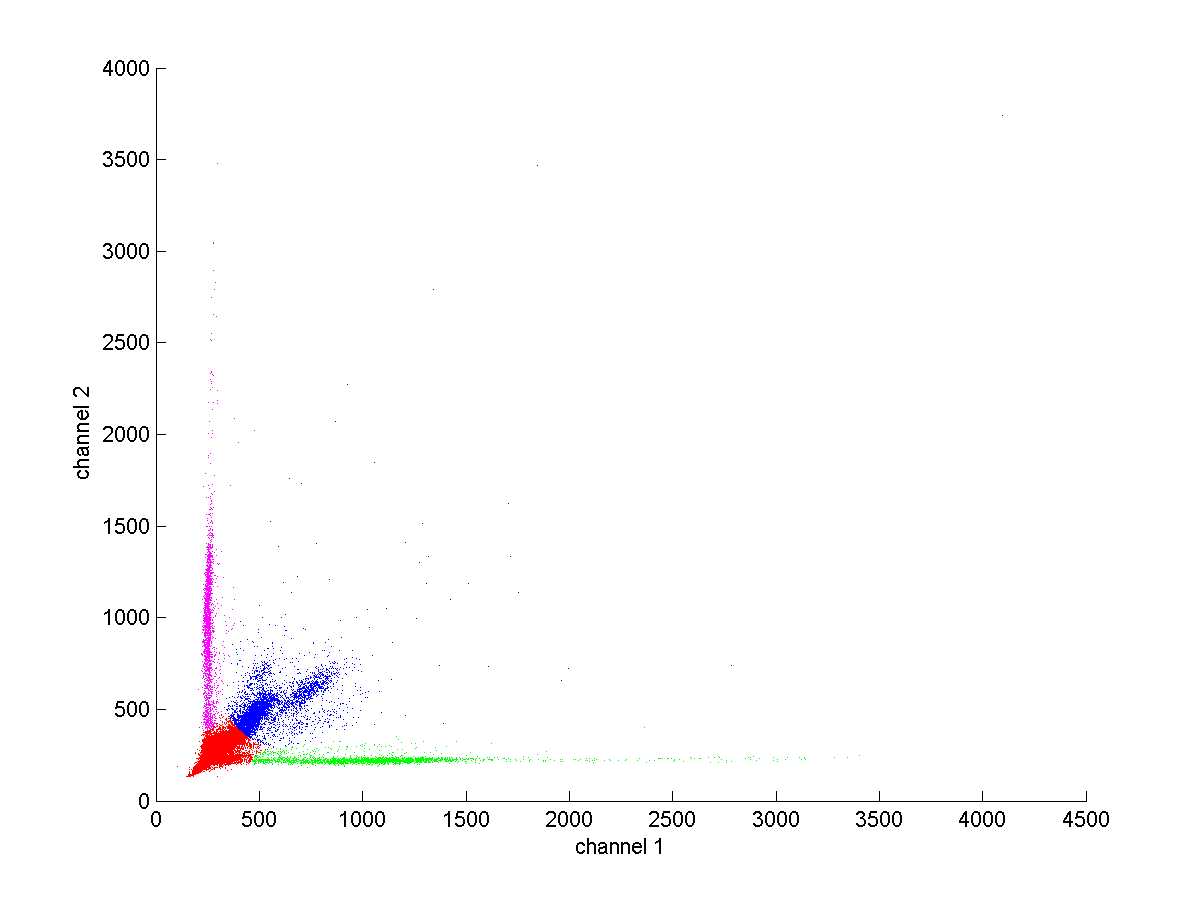

Supplement: Figure S3 — Distribution of average intensities of two fluorescent channels. The red, green, blue and pink cluster represent the 00, 10, 01, and 11 class respectively. It can be observed that it is difficult to delimit the 00 from the other 3 classes. (TIFF) [file pone.0036064.s004.tiff]

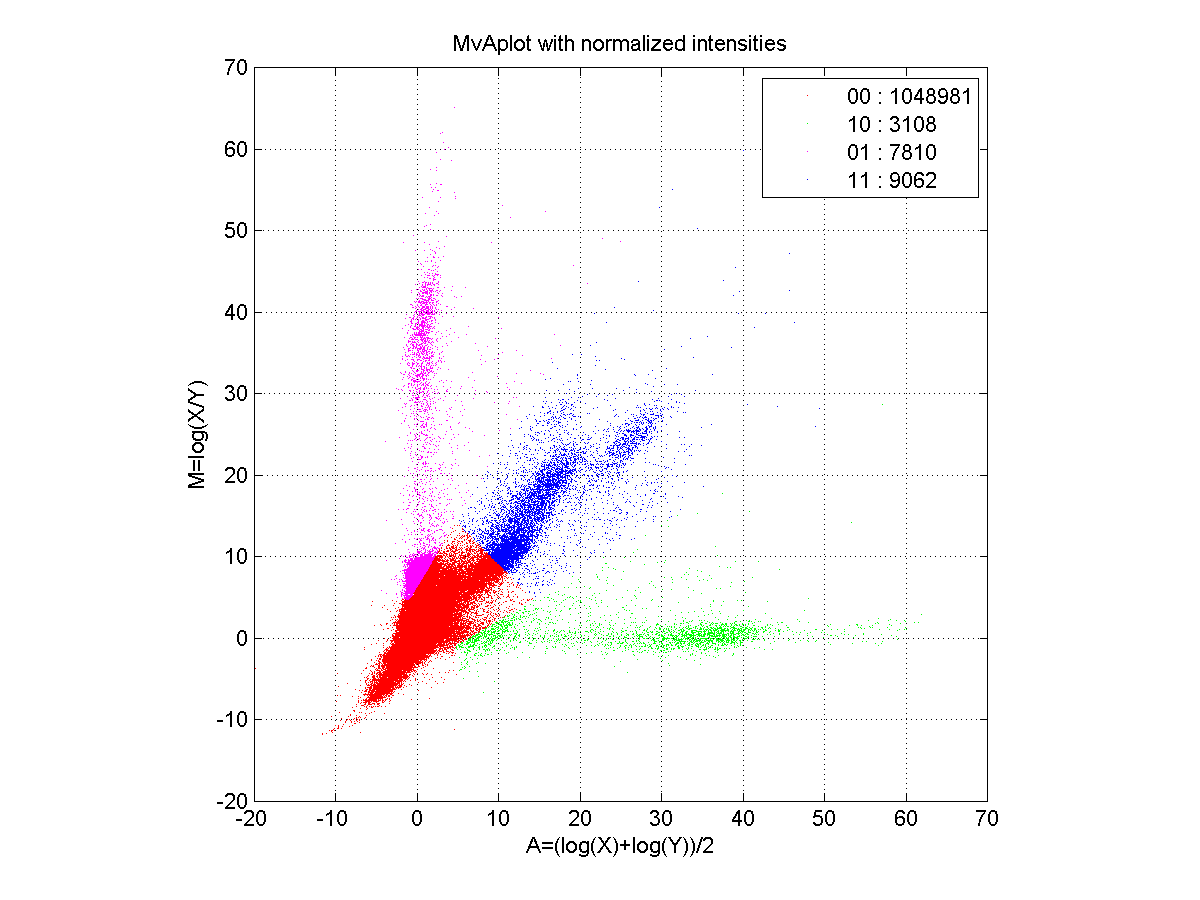

Supplement: Figure S4 — MvA plot with normalized intensities of two fluorescent channels. (TIFF) [file pone.0036064.s005.tiff]

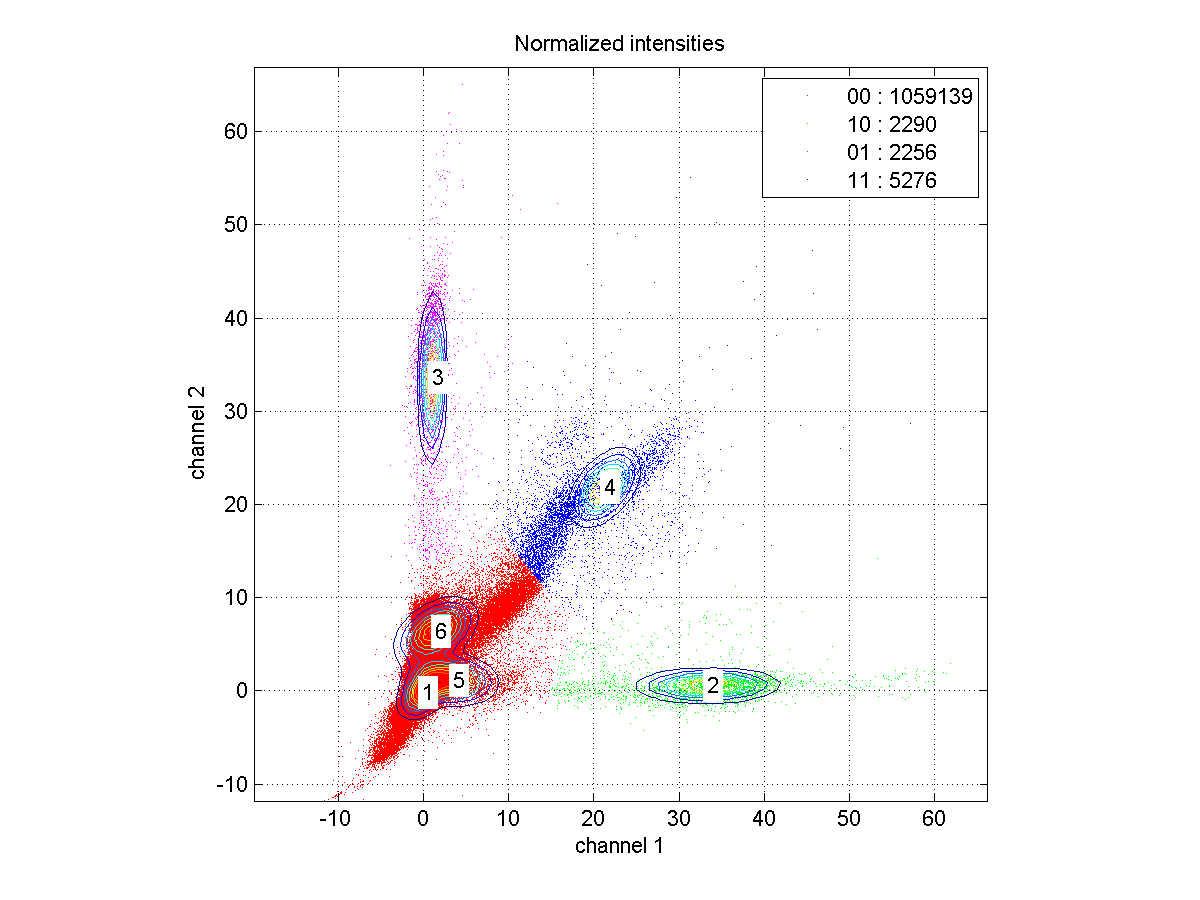

Supplement: Figure S5 — Normalized intensities of two fluorescent channels with the number of beads estimated for each of the four clusters. (TIFF) [file pone.0036064.s006.tiff]
