# Supplementary material for: Massively Parallel Haplotyping on Microscopic Beads for the High-Throughput Phase Analysis of Single Molecules
Source: PLoS One. 2012 Apr 30;7(4):e36064. doi: 10.1371/journal.pone.0036064 (PMC3340404; doi:10.1371/journal.pone.0036064)
Supplement: Table S1 — Target loss measured in the amplification of two targets using different template lengths and SNP combinations. 1Number of beads counted for a specific sequence string. The “0” in the sequence string represents an empty position for which only background florescence was recorded. Beads positives for more than one allele (two alleles per SNP) derived from multi-template reactions were removed from the data. 2Ratio of alleles obtained for heterozygous DNA 3Sum of beads informative for the queried SNPs relative to the total number of beads with a product. (DOCX) [file pone.0036064.s007.docx]

|  | Number of beads^1^ | Allelic ratio^2^ | Informative beads^3^ |
| --- | --- | --- | --- |
| **550 bp** |  |  |  |
| GT-beads | 66980 | 1.00 | 79.1% |
| AC-beads | 67271 |  |  |
| A/G0-beads | 22962 |  | 13.5% |
| 0C/T-beads | 12535 |  | 7.4% |
| **2500 bp** |  |  |  |
| GT-beads | 25326 | 1.06 | 91.6% |
| AC-beads | 26739 |  |  |
| A/G0-beads | 3288 |  | 5.8% |
| 0C/T-beads | 1513 |  | 2.7% |
| **5000 bp** |  |  |  |
| GT-beads | 2219 | 1.03 | 80.5% |
| AC-beads | 2291 |  |  |
| A/G0-beads | 475 |  | 8.5% |
| 0C/T-beads | 619 |  | 11.0% |
| **422 bp** |  |  |  |
| GC-beads | 1,576 | 1.06 | 81.6% |
| AT-beads | 1,490 |  |  |
| A/G0-beads | 260 |  | 6.9% |
| 0C/T-beads | 431 |  | 11.5% |
| **1220 bp** |  |  |  |
| TC-beads | 5,512 | 1.10 | 74.6% |
| CT-beads | 6,054 |  |  |
| A/G0-beads | 2530 |  | 16.3% |
| 0C/T-beads | 1418 |  | 9.1% |
| **2250 bp** |  |  |  |
| CC-beads | 4,837 | 1.05 | 65.1% |
| TT-beads | 5,083 |  |  |
| C/T0-beads | 3520 |  | 23.1% |
| 0C/T-beads | 1793 |  | 11.8% |
