# Supplementary material for: Massively Parallel Haplotyping on Microscopic Beads for the High-Throughput Phase Analysis of Single Molecules
Source: PLoS One. 2012 Apr 30;7(4):e36064. doi: 10.1371/journal.pone.0036064 (PMC3340404; doi:10.1371/journal.pone.0036064)
Supplement: Table S2 — Mutation rate of 4 different commercially available thermostable polymerases. The data was obtained amplifying a 422 bp template over 35 cycles that was then assayed by BEH targeting two loci. The fraction of mutants was estimated based on the number of beads with a thymidine instead of the expected cytidine. The error rate per cycle was estimated based on 35 cycles. (DOCX) [file pone.0036064.s008.docx]

| Enzyme | Haplotypes | fraction of mutants | Error rate per cycle | Haplotype ratio | Reported values | Ref |
| --- | --- | --- | --- | --- | --- | --- |
| Phusion | GC | 1.04E-05 | 2.98E-07 | 1.03 | 4.20E-07 | Li et al 2006 |
|  | AC |  |  |  |  |  |
|  | GT |  |  |  |  |  |
|  | AT |  |  |  |  |  |
| Phire | GC | 2.18E-04 | 6.22E-06 | 1.02 | 5.00E-06 | NEB reported rate |
|  | AC |  |  |  |  |  |
|  | GT |  |  |  |  |  |
|  | AT |  |  |  |  |  |
| Platinum | GC | 9.52E-04 | 2.72E-05 | 1.00 | 2.30E-05 | Li et al 2006 |
|  | AC |  |  |  |  |  |
|  | GT |  |  |  |  |  |
|  | AT |  |  |  |  |  |
| Titanium | GC | 1.78E-04 | 5.09E-06 | 1.12 | 1.00E-05 | Finnzymes reported rate |
|  | AC |  |  |  |  |  |
|  | GT |  |  |  |  |  |
|  | AT |  |  |  |  |  |
