# Supplementary material for: Massively Parallel Haplotyping on Microscopic Beads for the High-Throughput Phase Analysis of Single Molecules
Source: PLoS One. 2012 Apr 30;7(4):e36064. doi: 10.1371/journal.pone.0036064 (PMC3340404; doi:10.1371/journal.pone.0036064)
Supplement: Table S3 — Chimera formation in different product lengths. Amplicons were produced using Phusion in 35 cycles of PCR. (DOCX) [file pone.0036064.s009.docx]

| Enzyme | Haplotypes | No of beads | fraction of mutants | Haplotype ratio |
| --- | --- | --- | --- | --- |
| 550bp (Phusion) | AC | 21,445 | 3.72E-01 | 1.00 |
|  | GT | 21,443 |  |  |
|  | GC | 12,743 |  |  |
|  | AT | 12,702 |  |  |
| 801bp (Phusion) | TT | 23,093 | 2.00E-01 | 1.16 |
|  | CC | 26,801 |  |  |
|  | TC | 6,069 |  |  |
|  | CT | 6,436 |  |  |
| 422bp (Phire) | GC | 1,576 | 1.18E-01 | 1.06 |
|  | AT | 1,490 |  |  |
|  | AC | 217 |  |  |
|  | GT | 192 |  |  |
| 1349bp (Phire) | GT | 17,492 | 2.52E-01 | 1.08 |
|  | AC | 16,214 |  |  |
|  | GC | 4,199 |  |  |
|  | AT | 7,156 |  |  |
| 2500bp (Phusion) | AC | 26,739 | 7.55E-02 | 1.06 |
|  | GT | 25,326 |  |  |
|  | GC | 2,121 |  |  |
|  | AT | 2,129 |  |  |
| 5000bp (Phusion) | AC | 2,291 | 9.27E-02 | 1.03 |
|  | GT | 2,219 |  |  |
|  | GC | 150 |  |  |
|  | AT | 311 |  |  |
